# Further Support for the Psychometric Properties of the Farsi Version of Perth Alexithymia Questionnaire

**DOI:** 10.3389/fpsyg.2021.657660

**Published:** 2021-04-14

**Authors:** Arezou Lashkari, Mohsen Dehghani, Vahid Sadeghi-Firoozabadi, Mahmood Heidari, Ali Khatibi

**Affiliations:** ^1^Department of Psychology, Shahid Beheshti University, Tehran, Iran; ^2^Centre of Precision Rehabilitation for Spinal Pain (CPR Spine), School of Sport, Exercise and Rehabilitation Sciences, College of Life and Environmental Sciences, University of Birmingham, Birmingham, United Kingdom; ^3^Centre for Human Brain Health, University of Birmingham, Birmingham, United Kingdom

**Keywords:** alexithymia, psychometric properties, validity, reliabiity, Farsi (Persian)

## Abstract

Alexithymia is defined as the lack of words to describe emotions and is associated with different psychopathologies. Various tools have been developed for measuring alexithymia; each has its limitations. A new questionnaire, Perth Alexithymia Questionnaire (PAQ), was developed to simultaneously assess positive and negative dimensions. Validation of such a tool in different cultures allows cross-cultural health psychology studies and facilitates knowledge transfer in the field. We aimed to examine the psychometric features of the PAQ in the Farsi-speaking population in Iran. Four-hundred-twenty-nine university students were asked to complete the PAQ, the Toronto Alexithymia Scale (TAS-20), Beck Depression Inventory (BDI-II), Beck Anxiety Inventory (BAI), and emotion regulation questionnaire (ERQ). Concurrent validity, discriminant validity, internal consistency, and test-retest reliability and factor structure were investigated. Confirmatory factor analysis showed a five-factor model identical to the original questionnaire. The questionnaire indicated good internal consistency (0.82 < α < 0.94). Test-retest reliability was acceptable for all subscales. The correlations between PAQ and its subscales with BDI-II, BAI, and TAS, and expression suppression subscale of ERQ were strong for concurrent validity. Concerning the discriminant validity, PAQ and its subscales were not correlated with reappraisal subscales of ERQ. The present findings suggest that the Farsi version of PAQ has strong psychometric properties and is appropriate for use in the Farsi-speaking population.

## Introduction

Sifneos (1973) introduced alexithymia by describing psychosomatic patients who could not find appropriate words to express their emotions. Psychosomatic disorders have been described in various forms in previous versions of the Diagnostic and Statistical Manual of Mental Disorders (DSM). DSM-I described psychosomatic disorders in a section called psychophysiological autonomic and visceral disorders. DSM 5 refers to psychosomatic disorders in Somatic Symptom and Related Disorders (Moldovan et al., 2015). In all disorders of somatic symptom and related disorders, physical symptoms along with distress and impairment are prominent (American Psychiatric and Association [APA]., 2013). Alexithymia is one of the salient features of psychosomatic disorders. These patients show somatic symptoms instead of expressing emotions. Although alexithymia was coined first as a standard feature in psychosomatic disorders, it is prevalent in the normal population (Honkalampi et al., 2000; Kokkonen et al., 2001). Most researchers proposed that alexithymia is related to psychological pathologies. For example, Honkalampi et al. (2001) stated that the severity of depression was associated with alexithymia. de Bruin et al. (2019) showed that substance use disorder (SUD) patients with post-traumatic stress disorder (PTSD) are more alexithymic than SUD patients without PTSD. Iskric et al. (2020) suggest that alexithymia is related to non-suicidal self-injury and suicidal ideation. Passardi et al. (2019) indicated that alexithymia played an essential role in facial emotion recognition of negative emotions in PTSD. In addition, McCallum et al. (2003) showed that alexithymia could affect the treatment outcomes by interfering with the improvement of general symptoms.

These studies demonstrated the importance of alexithymia as a construct. Toronto Alexithymia Scale (TAS) (Bagby et al., 1994) and Bermond-Vorst Alexithymia Questionnaire (BVAQ) (Vorst and Bermond, 2001) are the two most commonly used measures. TAS includes difficulty describing feelings (DDF), difficulty identifying feelings (DIF), and externally oriented thinking (EOT). TAS-20 is one of the first developed measures of alexithymia for both clinical and non-clinical samples. However, some researchers claimed that the EOT subscale of TAS does not have enough internal consistency (Kojima et al., 2001; Taylor et al., 2003; Bagby et al., 2020), and items load poorly on the related latent factor (Preece et al., 2018a, b). TAS correlated positively with negative affect measures and raised concern that TAS might assess distress rather than alexithymia construct (Preece et al., 2020). The other criticism of TAS is that it does not take the valence of emotions into account (Preece et al., 2018b).

Bermond-Vorst Alexithymia Questionnaire, another commonly used tool, assesses cognitive and affective dimensions of alexithymia. The cognitive dimension of alexithymia refers to the processing of emotions at a cognitive level. The affective dimension measures the subjective report of individual emotional experience. Emotionalizing and fantasizing characterize affective alexithymia. Alexithymia’s cognitive dimension involve analyzing, identifying, and verbalizing emotions (Bermond et al., 2007). Differentiating the two dimensions of alexithymia is considered controversial (Goerlich, 2018). Some studies have failed to show the ability to differentiate between the two dimensions (Watters et al., 2016). Some researchers argued that difficulty in emotionalizing is not a valid indicator of alexithymia, as it does not distinguish between negative and positive reactivity to emotions (Preece et al., 2017).

Perth Alexithymia Questionnaire (PAQ; Preece et al., 2018b) was developed to address the earlier tool’s limitations. PAQ is a 24-item questionnaire containing 5-subscales: positive DIF (P-DIF), negative DIF (N-DIF), positive DDF (P-DDF), negative DDF (N-DDF), and general EOT (G-EOT). PAQ is developed based on the attention-appraisal model of emotions (Gross, 2015). The model contains four stages: situation, attention, evaluation, and response. When an emotional response becomes the stimulus (situation stage) that is the target of valuation, the person pays attention to it (attention stage) and starts to evaluate it (appraisal). Then, the individual might respond to it (response stage). According to the model, alexithymic people cannot focus their attention on emotional response. Furthermore, individuals with high levels of alexithymia cannot evaluate the emotional response as what it is and what it means. As a result, DIF and DDF are indicators of the appraisal stage. The salient point is that the appraisal stage can consist of valence. Both DIF and DDF subscales of PAQ comprise positive and negative valences and used to calculate the difficulty in the appraisal feeling (DAF) composite subscale. By combining the 5-subscale, composite subscales can be produced. In this regard, positive difficulty appraising feeling (P-DAF) includes positive DIF and positive DDF. Negative difficulty appraising feeling (N-DAF) is the sum of negative DIF and negative DDF (Preece et al., 2018b).

As mentioned, PAQ is constructed based on the attention-appraisal model of emotions. Most studies revealed that alexithymia reflects a deficit in regulating emotions (Swart et al., 2009; Pandey et al., 2011). Also, it has been suggested that alexithymic individuals used more suppression and less reappraisal for emotion regulation (Swart et al., 2009). Cognitive reappraisal means cognitive changes in the affective influence of the emotion-eliciting situation. Expressive suppression inhibits emotional behavior. Cognitive reappraisal is assumed as an adaptive emotion regulation strategy. However, expressive suppression is indicative of a maladaptive emotion regulation strategy (Gross and John, 2003). It is shown that emotional expression is strongly related to the cultural context (Altarriba and Kazanas, 2017). So, it is crucial to assess the psychometric features of the measures in other cultures.

Research showed the role of culture in factorial structure (Fernández-Jiménez et al., 2013). TAS-20 and more recently PAQ are measures for assessing the alexithymia in Iran. As we know, EOT component of the Farsi version of TAS-20 has lower internal consistency (Besharat, 2008). Besides, other studies aimed at validating PAQ in Iran failed to recruit a big enough sample or to report confirmatory analysis (Heydari et al., 2020; Mousavi Asl et al., 2020). Accordingly, the current study aimed to examine the validity and reliability of PAQ in Iran with specific reference to its internal consistency, test-retest reliability, concurrent and discriminant validity, and factor structure. In this regard, depression, anxiety inventories and TAS, suppression subscale of emotion regulation questionnaire (ERQ) were used for assessing concurrent validity. Discriminant validity was assessed by the cognitive reappraisal subscale of the ERQ. Confirmatory factor analyses of PAQ tested the construct validity. Considering the psychometric properties of the original version of the PAQ, we expected the measure to be highly reliable. We hypothesized that PAQ is an excellent measure to assess alexithymia as reflected in its correlation with other measures of emotional and mood difficulties. We also expected the PAQ to have a unique contribution above that of the previous measures as reflected in factor analysis.

### Method

#### Participants

Participants were recruited via advertisements on notice boards in the universities. They were included if they reported a good level of literacy, Including reading and understanding Farsi. Those with a history of traumatic experiences in the previous 6 months to the data collection point were excluded. A total of 436 individuals answered the questionnaires. Incomplete questionnaires were removed from the final sample leading to 429 (63.2% female, mean age = 21.35 ± 2.88, range = 18–39) unique responses that were included in the statistical analyses. All participants were university students, including 364 undergraduate, 41 masters, 11 Ph.D. students. The study was approved by the Research Ethics Committee of Shahid Beheshti University, Tehran, Iran.

## Materials and Methods

### Perth Alexithymia Questionnaire

Perth Alexithymia Questionnaire (Preece et al., 2018b) consists of 24 self-report measures rated on a seven-point Likert scale (1 = strongly disagree; 7 = strongly agree), with high scores demonstrating high levels of alexithymia. PAQ, the only alexithymia measure including the valence of emotions, consists of five subscales: negative-difficulty identifying feelings (N-DIF), positive-difficulty identifying feelings (P-DIF), negative-difficulty describing feelings (N-DDF), positive-difficulty describing feelings (P-DDF), and general-externally orientated thinking (G-EOT). “When I am feeling bad, I can’t tell whether I’m sad, angry or scared” and “when I’m feeling good, I get confused about what emotion it is” are the samples of N-DIF and P-DIF. The subscales can combine and produce the composite subscales. PAQ demonstrated good concurrent and discriminant validity and good internal consistency (Cronbach’s alpha of the subscales ranged from 0.87 to 0.96) (Preece et al., 2018b).

We followed a standard translation and back-translation procedure for the introduction of the Farsi version of the PAQ. First, the original questionnaire was translated into Farsi, and then a bilingual professional psychologist back-translated it into English. The back-translated version was compared with the original English one, a few minor corrections were applied and the final version of the PAQ, used in this study, was reached.

### 20-Item Toronto Alexithymia Scale

20-Item Toronto Alexithymia Scale (Bagby et al., 1994) is a 20-item measure consisting of three subscales: difficult identifying feelings (DIF), DDF, and EOT style. Each item is rated on a 5-point Likert scale (1 = strongly disagree to 5 = strongly agree). “When I am upset, I don’t know if I am sad, frightened or angry”, “It is difficult for me to find the right words for my feelings”, and “Being in touch with emotions is essential” are the samples of DIF, DDF, and EOT, respectively. Five key items are reverse scored (4, 5, 10, 118, and 19). TAS-20 demonstrated good internal consistency (Cronbach’s alpha ranged from 0.66 to 0.75) and test-retest reliability (0.77). The three-factor structure was congruent with the theoretical model underlying TAS-20 (Bagby et al., 1994). TAS psychometric properties have been tested and confirmed by various studies (Bressi et al., 1996; Taylor et al., 2003; Säkkinen et al., 2007). The Farsi version of TAS demonstrated good validity and reliability in the Farsi-speaking population (Besharat, 2008).

### Emotion Regulation Questionnaire

Emotion regulation questionnaire (Gross and John, 2003) is a short questionnaire designed to separate two subscales: expressive suppression (I keep my emotions to myself) and cognitive reappraisal (When I want to feel more positive emotion [such as joy or amusement], I change what I’m thinking about). ERQ is answered on a 7-point Likert scale (1 = strongly disagree, 7 = strongly agree). The Cronbach’s alpha was found to be in the range of 0.68–0.82 in different populations. ERQ demonstrated good convergent and discriminant validity (Gross and John, 2003). ERQ showed good validity and reliability in the Farsi-speaking population. The Cronbach’s alpha coefficient for cognitive reappraisal and expressive suppression were 0.78 and 0.60, respectively (Ghasempour et al., 2012).

### Beck Depression Inventory-II

Beck depression inventory-II (Beck et al., 1996) is a widely used measure for assessing depression in both clinical and non-clinical populations. BDI-II contains 21 items, each answer being scored on a Likert scale value of 0–3 in which higher scores indicate the existence of depression symptoms. One of the scale items is as follows: I do not feel sad, I feel sad much of the time, I am sad all the time, I am so sad or unhappy that I can’t stand it. Individuals are asked to select one of four possible items in each question based on the last 2 weeks state. The internal consistency is reported around 0.90, and test-retest reliability from 0.73 to 0.96. BDI-II demonstrated two-factor of cognitive-affective and somatic-vegetative (Wang and Gorenstein, 2013). In the Farsi-speaking sample, the same two-factor model is confirmed (Ghassemzadeh et al., 2005), and internal consistency and test-retest reliability were 0.87 and 0.74.

### Beck Anxiety Inventory

The BAI (Beck et al., 1988) is a 21-item self-report questionnaire that asks about common anxiety symptoms such as being scared, nervous, and unsteady. The items are rated on a 4-point Likert scale (0 = not at all, 3 = severely). The total score ranged from 0 to 63. The higher scores indicate a higher level of anxiety. BAI has the clinical classification, with scores from 0 to 7 displaying minimal anxiety, 8–15 as mild anxiety, 16–25 as moderate anxiety, and 26–63 as severe anxiety. Scores greater than 16 indicate clinically significant anxiety (Beck et al., 1988). BAI internal consistency (alpha Cronbach) and test-retest reliability reported as 0.91 and 0.65, respectively. BAI demonstrated a two-factor solution and concurrent validity with other anxiety measures and the BDI-II as well (Bardhoshi et al., 2016). In the Iranian population, the internal consistency coefficient (alpha Cronbach) was around 0.90, and the three-factor model is confirmed (Dobson and Mohamadkhani, 2008).

### Data Analysis

Confirmatory factor analyses (CFA) was performed using LISREL 8.80. All other analyses, such as correlations and descriptive statistics, were done by SPSS 24. As mentioned before, the PAQ concept is based on the attention-appraisal model of alexithymia. As a result, the subscale of PAQ can combine and produce composite subscales according to the theory (Preece et al., 2018b). Five theoretical models for the factor structure have been suggested. In this study, CFA was applied for all five models. CFA was conducted using the maximum likelihood estimation based on the Pearson covariance matrix. Maximum likelihood requires that the data display both univariate and multivariate normality, despite the robustness to violation of normality as long as the sample size is large (Gerbing and Anderson, 1985; Finney and DiStefano, 2006).

The fitness of the model was evaluated with the most important indices such as root mean square error of approximation (RMSEA), comparative fit index (CFI), normed fit index (NFI), incremental fit index (IFI), standard root mean squared residual (SRMR), Akaike information criterion (AIC), and relative chi-square (χ^2^/d*f*). Acceptable fit values of CFI, NFI, and IFI are greater than 0.90, while the acceptance value of χ^2^/d*f* is less than 5 (Hu and Bentler, 1999; Hooper et al., 2008). The acceptable value of RMSEA and SRMR is less than 0.08 and 0.1, respectively (Schumacker and Lomax, 2016). AIC is a criterion to compare different models, and lower values demonstrate better fitness (Kline, 2015).

Cronbach’s alpha was calculated to assess internal consistency. The values greater than 0.90 indicate excellent consistency, while those greater than 0.80 and 0.70 indicate good and acceptable internal consistency, respectively (Groth-Marnat, 2009). The test-retest reliability was examined in a separate group of 59 participants (76.3% female) with a mean age of 27.49 (SD = 5.12, range = 18–47) that were recruited only for this purpose. They completed the PAQ questionnaire two times with a 2-week interval between them. The test-retest reliability was quantified using a Pearson correlation estimate for total scores.

The Pearson correlation coefficient was used to assess concurrent and discriminant validity. We hypothesized that a medium to large (around 0.3 and 0.5) correlation (Cohen, 1998) between PAQ and TAS, BDI-II, BAI, and expressive suppression subscale of ERQ indicates concurrent validity. We expected a small, around 0.1 (Cohen, 1998) and even negative correlation of PAQ and cognitive reappraisal subscale of ERQ as discriminant validity.

### Procedure

At the beginning of the session, participants received the consent form. After reading and accepting to participate, they received the battery of questionnaires and were instructed to complete them by paying attention to the instruction on top of each questionnaire. The researcher (AL) was accessible during the time they were completing the questionnaires. At the end of the session, participants were debriefed. For the test-retest, due to the restriction imposed as the consequence of the COVID19 pandemic, a separate group of participants received a link with the online format of the translated PAQ questionnaire. They received a similar link 2 weeks later with the same questionnaire and were asked to answer it within 24 h.

## Results

### Descriptive Statistics

Table 1 presents the mean and standard deviation for subscales and total PAQ, and other measures in the study. The minimum and maximum mean and standard deviation belonged to the P-DIF and the G-EOT subscales, respectively (*M* = 9.84, SD = 4.96; *M* = 21.38, and SD = 9.57).

**TABLE 1:** Descriptive statistics for the administered measures.

Measure/subscale		Total	
		M	SD
Perth Alexithymia Questionnaire	P-DIF	9.84	4.96
	N-DIF	12.40	5.91
	P-DDF	11.05	5.83
	N-DDF	13.32	6.49
	G-EOT	21.38	9.57
	G-DIF	22.25	9.96
	G-DDF	24.37	11.27
	N-DAF	25.72	11.72
	P-DAF	20.89	10.27
	G-DAF	46.62	20.28
	ALEXI	68.01	27.05
TAS	DIF	15.71	5.69
	DDF	13.27	4.34
	EOT	18.94	3.90
ERQ	Expressive and suppression	13.23	5.42
	Cognitive and reappraisal	26.34	7.33
BAI		11.86	9.49
BDI-II		14.62	10.488

*M, mean; SD, standard deviation; PAQ, Perth Alexithymia Questionnaire; N-DIF, negative-difficulty identifying feelings; P-DIF, positive-difficulty identifying feelings; NDDF, negative-difficulty describing feelings; P-DDF, positive-difficulty describing feelings; G-EOT, general-externally orientated thinking; GDIF, general-difficulty identifying feelings; G-DDF, general-difficulty describing feelings; N-DAF, negative-difficulty appraising feelings; PDAF, positive-difficulty appraising feelings; DAF, general-difficulty appraising feelings; ALEXI, alexithymia; BAI, beck anxiety inventory; BDI, beck depression inventory; TAS, Toronto alexithymia questionnaire; DIF, difficulty identifying the feeling; DDF, difficulty describing the feeling; EOT, externally oriented thinking; and ERQ, emotion regulation questionnaire.*

### Confirmatory Factor Analyses

Before applying the CFA, all 24 items were checked for normal distribution. A skewness value of ±1 and ±2 is considered excellent and acceptable, respectively. While a value of ±3 is described as highly skewed. Kurtosis greater than 10 indicates non-normal distribution (George and Mallery, 2006; Kline, 2015). In the present study, the skewness ranged between 0.24 and 1.52, and the range of kurtosis was from −1.15 to 1.47, indicating a normal distribution of the scores.

**FIGURE 1:**
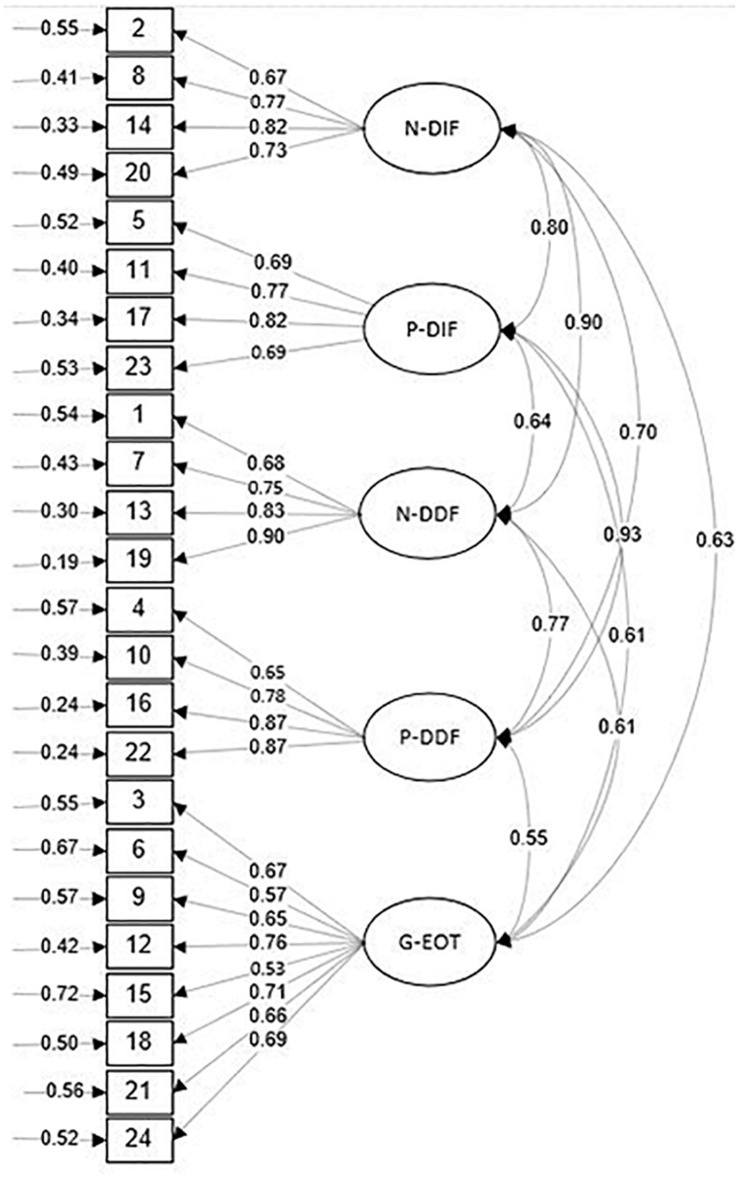
Confirmatory factor analysis: Item loadings on the 5-factor model, negative-difficulty identifying feelings (N-DIF), positive-difficulty identifying feelings (P-DIF), negative-difficulty describing feelings (N-DDF), positive-difficulty describing feelings (P-DDF), and general-externally orientated thinking (G-EOT).

Model 1 was a one-factor model in which all 24 items were loaded in general alexithymia. Model 2 was a two-factor model in which items were loaded on G-EOT and G-DAF. This model discriminates attention and appraisal stages of emotion evaluation. Model 3 was a three-factor model consisting of G-DIF, G-DDF, and G-EOT. G-DIF is obtained by combining positive and negative DIF, and the G-DDF is obtained by summing the positive DDF and negative DDF. There was no distinction between the valence of components in this model. Model 4 is a three-factor model based on the distinction of valence and includes G-EOT, N-DAF, and P-DAF factors. N-DAF is referred to as negative DIF, and negative DDF and P-DAF are created by collecting positive DIF and positive DDF. Model 5 is a five-factor model, and all factors were divided based on valence, DIF, and DDF. The items were loaded on G-EOT, N-DIF, P-DIF, N-DDF, and P-DDF. The goodness of fit values in Table 2 indicates that models 5 and 1 revealed the best and poorest fit indices, respectively. All the values of model 5 demonstrate that model 5 is the best-fitted model comparing other models. All items were loaded well on five hypothesized factors (Figure 1).

**TABLE 2:** Goodness-of-fit index values from confirmatory factor analysis of the 24 Perth Alexithymia Questionnaire items.

Model	Chi-Square	Df	χ2/d*f*	RMSEA	CFI	NFI	IFI	AIC	SRMR
Model1	2,857.29	252	11.33	0.15	0.91	0.90	0.91	2,953.29	0.09
Model2	1,892.36	251	7.53	0.12	0.94	0.93	0.94	1,990.36	0.06
Model3	1,928.67	249	7.74	0.12	0.94	0.93	0.94	2,030.15	0.06
Model4	10.56	249	4.24	0.08	0.97	0.95	0.97	1,158.67	0.04
Model5	784.53	242	3.24	0.07	0.97	0.96	0.97	900.53	0.04

*Df, the degree of freedom; χ^2^/df, relative chi-square; RMSEA, root mean square error; CFI, the comparative fit index; NFI, normed fit index; IFI, incremental fit index; SRMR, standard root mean squared residual; and AIC, Akaike information criterion.*

### Reliability

We examined the reliability of the measure in two different ways. First, we measured the test-retest reliability for the subscales and total scales, which are presented in Table 3 (left column). Test-retest reliability of all subscales was statistically significant after at least 2 weeks (*r* > 0.69). Cronbach’s alpha coefficient ranged from 0.82 to 0.94 for subscales and 0.94 for the total score, suggesting the good internal consistency of PAQ.

**TABLE 3:** Test-retest reliability and Cronbach’s alpha reliability coefficients for administered measures.

Measure/subscales		Test-retest correlation	Cronbach’s alpha (α)
PAQ	P-DIF	0.71**	0.82
	N-DIF	0.72**	0.83
	P-DDF	0.69**	0.87
	N-DDF	0.71**	0.87
	G-EOT	0.74**	0.85
	G-DIF	0.76**	0.88
	G-DDF	0.77**	0.90
	N-DAF	0.75**	0.91
	P-DAF	0.76**	0.91
	G-DAF	0.81**	0.94
	ALEXI	0.85**	0.94

PAQ, Perth Alexithymia Questionnaire; N-DIF, negative-difficulty identifying feelings; P-DIF, positive-difficulty identifying feelings; NDDF, negative-difficulty describing feelings; P-DDF, positive-difficulty describing feelings; G-EOT, general-externally orientated thinking; GDIF, general-difficulty identifying feelings; G-DDF, general-difficulty describing feelings; N-DAF, negative-difficulty appraising feelings; PDAF, positive-difficulty appraising feelings; DAF, general-difficulty appraising feelings; ALEXI, alexithymia. **p < 0.01.

### Concurrent and Discriminant Validity

Table 4 presents the correlation of all subscales of PAQ and TAS, ERQ, BAI, and BDI-II. As expected, the correlations between all subscales of PAQ and TAS are statistically significant, ranging from 0.18 to 0.73. Also, there are positive correlations between PAQ and BDI-II and BAI. The expressive suppression subscale of ERQ and PAQ subscales were significantly correlated (0.37–0.53), and no significant correlation was found between PAQ and cognitive reappraisal.

**TABLE 4:** Pearson correlations between the Perth Alexithymia Questionnaire, beck depression inventory, beck anxiety inventory, emotion regulation questionnaire, and Toronto Alexithymia Scale.

	Measures/subscales	BDI-II	BAI	ERQ		TAS-		
				
				Cognitive reappraisal	Expressive suppression	DIF	DDF	EOT
PAQ	Subscales							
	N-DIF	0.39**	0.33**	0.03	0.39**	0.73**	0.57**	0.18**
	P-DIF	0.28**	0.21**	−0.04	0.37**	0.62**	0.50**	0.28**
	N-DDF	0.35**	0.20**	0.01	0.44**	0.58**	0.67**	0.19**
	P-DDF	0.26**	0.16**	−0.07	0.41**	0.52**	0.58**	0.22**
	G-EOT	0.34**	0.21**	0.02	0.53**	0.47**	0.49**	0.28**
	Composites							
	G-DIF	0.37**	0.30**	−0.01	0.41**	0.74**	0.59**	0.25**
	G-DDF	0.34**	0.20**	0.00	0.46**	0.61**	0.69**	0.22*
	N-DAF	0.39**	0.28**	0.02	0.44**	0.69**	0.66**	0.20**
	P-DAF	0.28**	0.19**	−0.02	0.41**	0.60**	0.58*	0.26**
	G-DAF	0.37**	0.26**	−0.04	0.46**	0.70**	0.67**	0.25**
	ALEXI	0.40**	0.27**	−0.01	0.53**	0.70**	0.68**	0.28**

PAQ, Perth Alexithymia Questionnaire; N-DIF, negative-difficulty identifying feelings; P-DIF, positive-difficulty identifying feelings; NDDF, negative-difficulty describing feelings; P-DDF, positive-difficulty describing feelings; G-EOT, general-externally orientated thinking; GDIF, general-difficulty identifying feelings; G-DDF, general-difficulty describing feelings; N-DAF, negative-difficulty appraising feelings; PDAF, positive-difficulty appraising feelings; DAF, general-difficulty appraising feelings; ALEXI, alexithymia; BAI, beck anxiety inventory; BDI, beck depression inventory; TAS, Toronto alexithymia questionnaire; DIF, difficulty identifying the feeling; DDF, difficulty describing the feeling; EOT, externally oriented thinking; and ERQ, emotion regulation questionnaire. **(p < 0.01); *(p < 0.05).

## Discussion

This study aimed to investigate the utility of PAQ in Farsi-speaking people living in Iran. We aimed to determine which of the original model’s five variants would fit best in the Farsi-speaking population. Although most of the fit indices were acceptable in all five models, model 5 (which consists of five-factors) was the best. In model 5, all 24 items were loaded on the related factors. Therefore, it can be claimed that the Farsi version of the PAQ is a multidimensional questionnaire. This claim is consistent with the results of Preece et al. (2018b). As assumed by prior studies (Barrett et al., 2001), differences between positive and negative emotions are important for emotion regulation because the individuals differ in their emotional experiences. One of the most distinctive features of the PAQ is that it considers the valence of emotions, which is well described in separating the positive and negative valence in apprising factors. P-DIF, N-DIF, P-DDF, and N-DDF are the indicators of the appraisal stage of the model. Model 5 contains all four mentioned factors plus the EOT, which is not valence specified.

Testing the measure’s reliability, we found the PAQ subscale and the composite subscales to be reliable constructs similar to the original version (Preece et al., 2018b). The purpose of creating the new measure was to overcome the limitations of previous tools, including the low reliability of the EOT subscale. Most studies presented that EOT component has low reliability in different cultures (Bressi et al., 1996; Kojima et al., 2001; Taylor et al., 2003). In the present study, all the alpha coefficients were greater than 0.80, reflecting good internal consistency, particularly EOT subscale, which is consistent with the study by Preece et al. (2018b). The original study did not assess the test-retest reliability (Preece et al., 2018b). All subscales, especially the total alexithymia, indicated good test-retest reliability, which means that the scale results are consistent at different time points. The test-retest reliability results in the present study are comparable with those reported by Mousavi Asl et al. (2020).

To test the concurrent and discriminant validity of the PAQ, we measured the correlations between the subscales and other previously validated measures, including the BDI-II, BAI, TAS, and ERQ. In agreement with the original study (Preece et al., 2018b), PAQ was strongly correlated with all TAS subscales, which is another alexithymia measure. A score for general difficulties in identifying and describing feelings will be obtained by combining the negative and positive subscales. These two subscales, along with the externally orientated thinking subscale, are the same subscales that TAS measures too. Therefore, a high correlation between the two measures was expected. The correlation of PAQ with BDI-II and BAI is in line with the original study (Preece et al., 2018b). Individuals with depression and anxiety seemed to have difficulty recognizing and describing their emotions. Honkalampi et al. (2001) stated that alexithymia is associated with depression. Moreover, Honkalampi et al. (2000) reported that the prevalence of alexithymia in individuals with mild depression (BDI > 9) was 32%. Another study showed that difficulties in describing and identifying feelings, changes with mood and recovery from depression and were associated with a decrease in alexithymia (Saarijärvi et al., 2001). Alexithymia is also associated with anxiety. Difficulties describing and identifying feelings are related to anxiety disorders such as generalized anxiety disorder (GAD), and the presence of alexithymia is related to higher levels of anxiety (Berardis et al., 2008).

The expressive suppression subscale of the ERQ was correlated positively with the PAQ subscales. However, PAQ was not correlated with the cognitive reappraisal subscale of ERQ. Cognitive reappraisal and expressive suppression are considered adaptive and maladaptive emotion regulation, respectively (Gross and John, 2003). As mentioned before, alexithymia is defined as a deficiency in expressing emotions. Accordingly, it is expected that alexithymia would be correlated with expressive suppression. In line with the lack of relationship between alexithymia and cognitive reappraisal, it can be stated that alexithymia is an obstacle to regulating emotions (Gross, 1998). It can be assumed that the alexithymia has concurrent and discriminant validity.

The present study, despite its advantages, also suffers from some limitations. Firstly, the study was carried out among university students. Therefore, generalizing the finding to other populations, especially clinical samples, should be done with caution. Secondly, the cutoff point was not assessed, and this will limit the use of the measure for clinical research. Hence, for future studies, it is recommended to evaluate the psychometric feature of the PAQ in other populations, especially clinical samples.

Understanding emotion and its contribution to wellbeing is important in health psychology research. Because of the universality of emotions, they can help us in cross-cultural studies. Adaptation of the existing tools to new languages and in different cultures can facilitate cross-cultural health psychology research (Rudell and Diefenbach, 2008). PAQ (Preece et al., 2018b) is one of the most widely used tools to test alexithymia. Previous efforts in adapting and validating this tool among the Farsi-speaking population suffer from low sample size and lack of model estimation. In the current study, we examined the psychometric properties of a new adaptation of the PAQ (please see Supplementary Material) in a large Farsi-speaking sample in Iran. In summary, the study findings show that the Farsi version of PAQ is a valid and reliable measure. PAQ seems to be a promising measure for identifying the deficiency of emotions in the Farsi-speaking sample.

## Data Availability Statement

The raw data supporting the conclusions of this article will be made available by the authors, without undue reservation.

## Ethics Statement

The studies involving human participants were reviewed and approved by Department of Psychology, Shahid Beheshti University. The patients/participants provided their written informed consent to participate in this study.

## Author Contributions

AL was involved in design, data collection, analysis, and writing. MD and AK was involved in design and writing. VS-F and MH was involved in design. All authors contributed to the article and approved the submitted version.

## Conflict of Interest

The authors declare that the research was conducted in the absence of any commercial or financial relationships that could be construed as a potential conflict of interest.
