# Supplementary material for: Further Support for the Psychometric Properties of the Farsi Version of Perth Alexithymia Questionnaire
Source: Front Psychol. 2021 Apr 14;12:657660. doi: 10.3389/fpsyg.2021.657660 (PMC8079730; doi:10.3389/fpsyg.2021.657660)
Supplement: Supplementary file 1 [file Table_1.DOCX]

Perth Alexithymia Questionnaire-Farsi version

این پرسشنامه درباره این است که شما چه طور هیجاناتتان را تجربه و درک می‌کنید. لطفاً عبارات زیر را بر اساس اینکه **تا چه حد موافق یا مخالف این هستید که عبارت موردنظر در مورد شما صدق می‌کند** نمره‌گذاری کنید. برای هر عبارت یک پاسخ را انتخاب کنید. برخی سؤالات **به هیجانات بد یا ناخوشایند** اشاره دارند، این به معنی هیجاناتی مثل غمگینی، خشم و یا ترس است. برخی سؤالات به **هیجانات خوشایند یا مطلوب** اشاره دارند، منظور هیجاناتی مثل خوشحالی، سرگرمی و هیجان‌زده بودن است.

|  | کاملاً مخالف | ---- | --- | نه مخالف نه موافق | --- | --- | کاملاً موافق |
| --- | --- | --- | --- | --- | --- | --- | --- |
| 1. وقتی احساس بدی (احساس یک هیجان ناخوشایند) دارم، نمی‌توانم کلمات مناسب را برای توصیف آن احساسات پیدا کنم. | 1 | 2 | 3 | 4 | 5 | 6 | 7 |
| 1. وقتی احساس بدی دارم، نمی‌توانم بگویم که آیا غمگینم، عصبانیم و یا ترسیده‌ام. | 1 | 2 | 3 | 4 | 5 | 6 | 7 |
| 1. من معمولاً اینکه چه احساسی دارم را نادیده می‌گیرم. | 1 | 2 | 3 | 4 | 5 | 6 | 7 |
| 1. وقتی احساس خوبی (احساس یک هیجان خوشایند) دارم، نمی‌توانم کلمات مناسب را برای توصیف آن احساسات پیدا کنم. | 1 | 2 | 3 | 4 | 5 | 6 | 7 |
| 1. وقتی احساس خوبی دارم، نمی‌توانم بگویم که آیا شادم، هیجان‌زده‌ام و یا سرگرم شدم. | 1 | 2 | 3 | 4 | 5 | 6 | 7 |
| 1. من ترجیح می‌دهم به‌جای اینکه بخواهم روی احساساتم تمرکز کنم بگذارم پس ذهنم رخ دهند. | 1 | 2 | 3 | 4 | 5 | 6 | 7 |
| 1. وقتی احساس بدی دارم ، نمی‌توانم خیلی عمیق یا با جزییات راجع به آن‌ها حرف بزنم. | 1 | 2 | 3 | 4 | 5 | 6 | 7 |
| 1. وقتی احساس بدی دارم، نمی‌توانم از این احساسات سر دربیاورم. | 1 | 2 | 3 | 4 | 5 | 6 | 7 |
| 1. من به هیجاناتم توجهی نمی‌کنم. | 1 | 2 | 3 | 4 | 5 | 6 | 7 |
| 1. وقتی احساس خوبی دارم، نمی‌توانم خیلی عمیق یا با جزییات راجع به آن‌ها حرف بزنم. | 1 | 2 | 3 | 4 | 5 | 6 | 7 |
| 1. وقتی احساس خوبی دارم، نمی‌توانم از این احساسات سر دربیاورم. | 1 | 2 | 3 | 4 | 5 | 6 | 7 |
| 1. معمولاً سعی می‌کنم از فکر کردن درباره اینکه چه احساسی دارم، اجتناب کنم. | 1 | 2 | 3 | 4 | 5 | 6 | 7 |
| 1. وقتی اتفاق بدی می‌افتد، برایم سخت است اینکه چه احساسی دارم را در قالب کلمات بیان کنم. | 1 | 2 | 3 | 4 | 5 | 6 | 7 |
| 1. وقتی احساس بدی دارم، سردرگمم که دستخوش چه هیجانی هستم. | 1 | 2 | 3 | 4 | 5 | 6 | 7 |
| 1. ترجیح می‌دهم به‌جای تمرکز بر هیجاناتم، بر روی چیزهایی که درواقع می‌توانم ببینم یا لمس کنم تمرکز کنم. | 1 | 2 | 3 | 4 | 5 | 6 | 7 |
| 1. وقتی اتفاق خوبی می‌افتد، برایم سخت است اینکه چه احساسی دارم را در قالب کلمات بیان کنم. | 1 | 2 | 3 | 4 | 5 | 6 | 7 |
| 1. وقتی احساس خوبی دارم، سردرگمم که دستخوش چه هیجانی هستم. | 1 | 2 | 3 | 4 | 5 | 6 | 7 |
| 1. سعی نمی‌کنم که با هیجاناتم در تماس باشم. | 1 | 2 | 3 | 4 | 5 | 6 | 7 |
| 1. وقتی احساس بدی دارم، اگر بخواهم تلاش کنم تا احساسم را توصیف کنم، نمی‌دانم که چه باید بگویم. | 1 | 2 | 3 | 4 | 5 | 6 | 7 |
| 1. وقتی احساسات بدی دارم، آن‌ها مرا گیج می‌کنند. | 1 | 2 | 3 | 4 | 5 | 6 | 7 |
| 1. برایم مهم نیست که بدانم چه احساسی دارم. | 1 | 2 | 3 | 4 | 5 | 6 | 7 |
| 1. وقتی احساس خوبی دارم، اگر بخواهم تلاش کنم تا احساسم را توصیف کنم، نمی‌دانم که چه باید بگویم. | 1 | 2 | 3 | 4 | 5 | 6 | 7 |
| 1. وقتی احساسات خوبی دارم، آن‌ها مرا گیج می‌کنند. | 1 | 2 | 3 | 4 | 5 | 6 | 7 |
| 1. برایم عجیب است که درباره هیجاناتم فکر کنم. | 1 | 2 | 3 | 4 | 5 | 6 | 7 |
